# Identification of items for a standardised resource-use measure: review of current instruments

**DOI:** 10.1186/1745-6215-16-S2-O26

**Published:** 2015-11-16

**Authors:** Joanna Thorn, Colin Ridyard, Ruth Riley, Sara Brookes, Dyfrig Hughes, Sarah Wordsworth, Sian Noble, William Hollingworth

**Affiliations:** 1 University of Bristol, Bristol, UK; 2 Bangor University, Bangor, UK; 3 University of Oxford, Oxford, UK

## Background

Resource-use measurement by patient recall in economic evaluations alongside clinical trials is currently characterised by inconsistency and a lack of validation. A fully validated standardised resource-use measure (RUM) could potentially increase data quality, improve comparability between cost-effectiveness analyses and reduce research burden on health economists.

## Aim

To review the content of existing RUMs with a view to conducting a Delphi survey to identify core items that should be included in any UK trial-based economic evaluation.

## Methods

A single version of each instrument designed for use in a UK-based study was identified from the Database of Instruments for Resource-Use Measurement (http://www.dirum.org). Section headings (‘domains’) and questions (‘items’) were extracted verbatim according to a predefined schema. Information on the recall period, level of detail, use of skip logic and scope (disease-specific or total resource use) was also extracted. Items were scrutinised for overlap.

## Results

In excess of 2000 items were extracted from 59 instruments. The range of structures used to collect data was extremely wide, and varying levels of information were requested about similar items (e.g. hospital stays/nights). Recall periods varied substantially (sometimes within an instrument), and total resource use was more commonly requested than disease-specific resource use. Skip logic was employed in over half the instruments reviewed. The items were reduced to 350 following preliminary scrutiny for overlap.

## Preliminary conclusions

There is substantial variation in the methods used to assess resource use in clinical trials. Further work is in progress to prepare the items for inclusion in a Delphi survey.

